# The Molecular Weaponry Produced by the Bacterium *Hafnia alvei* in Foods

**DOI:** 10.3390/molecules27175585

**Published:** 2022-08-30

**Authors:** José Ramos-Vivas, Olga Tapia, María Elexpuru-Zabaleta, Kilian Tutusaus Pifarre, Yasmany Armas Diaz, Maurizio Battino, Francesca Giampieri

**Affiliations:** 1 Research Group on Foods, Nutritional Biochemistry and Health, Universidad Europea del Atlántico, 39011 Santander, Spain; 2 Research Group on Foods, Nutritional Biochemistry and Health, Universidad Internacional Iberoamericana, Campeche 24560, Mexico; 3 CIBER of Infectious Diseases—CIBERINFEC, Instituto de Salud Carlos III, 28029 Madrid, Spain; 4 Department of Clinical Sciences, Polytechnic University of Marche, 60131 Ancona, Italy; 5 International Joint Research Laboratory of Intelligent Agriculture and Agri-Products Processing, Jiangsu University, Zhenjiang 212013, China; 6 Department of Biochemistry, Faculty of Sciences, King Abdulaziz University, Jeddah 80200, Saudi Arabia

**Keywords:** *Hafnia alvei*, quorum-sensing, probiotics, biopreservation, foodborne pathogens

## Abstract

*Hafnia alvei* is receiving increasing attention from both a medical and veterinary point of view, but the diversity of molecules it produces has made the interest in this bacterium extend to the field of probiotics, the microbiota, and above all, to its presence and action on consumer foods. The production of Acyl Homoserine Lactones (AHLs), a type of quorum-sensing (QS) signaling molecule, is the most often-studied chemical signaling molecule in Gram-negative bacteria. *H. alvei* can use this communication mechanism to promote the expression of certain enzymatic activities in fermented foods, where this bacterium is frequently present. *H. alvei* also produces a series of molecules involved in the modification of the organoleptic properties of different products, especially cheeses, where it shares space with other microorganisms. Although some strains of this species are implicated in infections in humans, many produce antibacterial compounds, such as bacteriocins, that inhibit the growth of true pathogens, so the characterization of these molecules could be very interesting from the point of view of clinical medicine and the food industry. Lastly, in some cases, *H. alvei* is responsible for the production of biogenic amines or other compounds of special interest in food health. In this article, we will review the most interesting molecules that produce the *H. alvei* strains and will discuss some of their properties, both from the point of view of their biological activity on other microorganisms and the properties of different food matrices in which this bacterium usually thrives.

## 1. Introduction

*Hafnia alvei* is a Gram-negative bacillus that belongs to a family recently proposed as *Hafniaceae*, within the order *Enterobacterales* [1,2]. *H. alvei* occurs in many natural environments such as rivers and fish farms, as well as in polluted waters and sewage [3,4]. This bacterium is also common in the digestive tract of many animal [5]. Some *H. alvei* strains were considered as being an opportunistic pathogen in several animal species including mammals, fish, birds and insects [6], and are also suspected to cause a variety of disorders in humans [3,7]. In studies prior to 2010, the use of biochemical tests provided a rapid but imprecise identification of this species. However, in the last decade, the taxonomy of this species has been fully explained, which was differentiated from other closely related species with biochemical tests, as well as specific PCR and DNA–DNA hybridization tests, and through the sequencing of total or partial genomes [2,8,9]. These improvements in taxonomic identification have shown that some strains of *H. alvei* were actually strains of related species such as *Escherichia*, *Citrobacter*, *Obesumbacterium*, *Salmonella*, *Serratia*, or *Yokenella* [3,10,11,12]. In addition, researchers have also realized that *H. paralvei*, a very similar species, was being incorrectly classified as *H. alvei* by many laboratories [9]. In food microbiology, many laboratories continue to use traditional systems such as the API system, the Microbat system or the Biolog system, although more precise tests such as 16S rRNA gene sequencing or faster tests such as identification using MALDI–TOF MS are gradually being established. The problem when using MALDI–TOF comes mainly from updating its databases, since they are normally optimized for the identification of human pathogens, and not so much for the identification of environmental pathogens. All these systems were used to identify strains of *H. alvei* isolated from food with more or less satisfactory results [13,14]. It is highly desirable that the companies that manufacture these tools begin to incorporate the needs of food microbiologists and veterinarians into their databases.

Although some virulence traits have been studied in *H. alvei*, little is known about the factors that contribute to their pathogenesis within a host, including motility, surface appendages for adherence to eukaryotic cells, and biofilm formation [15,16,17]. The best-studied strains in terms of virulence mechanisms are human and fish isolates, although the range of hosts of this species is very wide [16,18]. At the moment, the number of strains included in these studies is very limited and we also detected a clear shortage of mechanistic studies that could complement descriptive studies. In addition, there is a lack of more in vitro studies with eukaryotic cells, and also in vivo models, with complex animals such as zebrafish or mice or simple ones such as the *Galleria mellonella* worm. In addition to its putative virulence factors and despite their apparently low virulence, predisposing factors such as immunodeficiency may favor some pathologies associated with *H. alvei* infection in humans. Importantly, some *H. alvei* strains isolated from different foods encode toxin genes such as the *E. coli* heat-stable toxin and a verotoxin [19]. Verotoxins in *H. alvei* were demonstrated in in vitro assays against a tissue culture cell line derived from monkey kidney epithelial cells [8].

Another emerging research topic is its resistance to antibiotics. Some *H. alvei* are resistant to important antibiotics such as some third-generation cephalosporins and carbapenems. Other studies indicate that it is a promiscuous species in terms of the acquisition or transference of resistance determinants by means of plasmids [20,21,22]. Antibiotic resistance data indicate that this bacterium is especially resistant to colistin, an antibiotic of last resort in human medicine, placing this bacterium in the group of bacteria that are naturally resistant to this antibiotic, such as *Proteus*, *Providencia*, and *Serratia* [21,22,23,24,25].

This fragmented knowledge about its biology at different levels has not prevented it from being an interesting bacterial species also from the point of view of the food industry as well. As we discussed above, its extensive distribution in nature also makes it a common inhabitant of the microbiota of many species of aquatic and terrestrial animals and may spread during slaughtering operations. As an example, Patterson and Gibbs reported this bacterium on carcasses, tables in the boning room, and on the hands of workers [26]. Consequently, this bacterium was isolated from meat, fish, and dairy products.

Some of the strains reported in the literature are clearly harmful to food preservation and to the full maintenance of their organoleptic properties. However, other potentially beneficial strains were reported, mainly to control the proliferation of other microorganisms with true pathogenic capacities such as *Salmonella* spp., *Bacillus* sp., or some serotypes of *E. coli*, including enterotoxigenic strains, among others. *H. alvei* was also included in a list of microorganisms with a desirable contribution to food fermentations [27]. Furthermore, in an interesting series of articles, French researchers have used a strain of *H. alvei* that appears to have interesting probiotic properties in obese mice. When introduced into the food of these obese mice, body weight, fat mass gain and tissue adiposity were reduced [28,29].

In this review, we examine the most interesting molecules present in *H. alvei* strains and discuss some of their properties, both from the point of view of their biological activity on other microorganisms and from the point of view of the food matrix, where this bacterium seems to act as an important agent of food spoilage in some cases. 

## 2. Genomics

The development of genome sequencing technologies and advanced computational analyses has increased our capacity for bacterial product discovery. Genome mining approaches identify those genes involved in secondary metabolite production, which can reveal the biosynthetic potential in many bacterial species. However, with the advent of genome sequence information, we also came across a lot of information, including the problem of being able to apply this information to biological questions. As of 1 August 2022, 35 *H. alvei* genomes were made publicly available in the GenBank database, of which only six are complete (available online: Genome List-Genome-NCBI (https://www.ncbi.nlm.nih.gov/genome/browse/#!/prokaryotes/10737/, accessed on 1 August 2022). Complete genomes belong to strains of different origins; thus, genomes of human clinical strains can be found (strains HUMV5920, PCM-1220 and CBA7135), one isolate from plant rhizosphere (strain A23BA), and one strain isolated from fish meat (strain FB1). The last one is an isolate from the human gut (sample name MGYG-HGUT-02508). The size of *H. alvei* genomes varies between 4.47 Mbp and 5.01 Mbp with an average size of 4.79 Mbp. Furthermore, the percentage of G + C was 49.58%, varying between 48.2% and 49%. Obviously, much information is still hidden in the uncompleted genomes, which would undoubtedly enable us to better understand the characteristics of this species and why some strains of *H. alvei* are predominant in some environments and not in others, or their ability to use some metabolites or others depending on the food matrix in which the strains are located. 

## 3. Quorum Sensing

Genomes from the sequenced environmental and pathogenic *H. alvei* strains contain genes for quorum sensing, mobility and biofilm formation [4,5,30]. The production of several enzymatic activities in *H. alvei* is under the control of quorum-sensing (Q-S) mechanisms [31], which also occur in other bacteria [32]. Quorum sensing enables bacteria to regulate their collective behavior depending on the population cell density in a certain environment, which is a mechanism that exists in many food spoilage bacteria. As with other Gram-negative food spoilage bacteria, *H. alvei* uses an array of N-homoserine lactones as signaling molecules for QS [33,34]. Homoserine lactones (HLs) or N-acyl homoserine lactones (AHLs) are molecules commonly produced by Gram-negative bacteria, including food-borne pathogens such as bacteria of the genus *Aeromonas* [35], *Shewanella* [36], *Clostridium* [37], *Pseudomonas* [38], and *Salmonella* [39] among others, which constitute a cell density-dependent intercellular signaling mechanism that modulates a great variety of physiological processes, including the production of different virulence factors. Many strains of *Enterobacteriaceae* isolated from food are able to synthesize AHLs because these molecules are easily detectable from contaminated foods using biosensor strains or biochemical assays. The production of these molecules is also common among proteolytic psychrotrophic bacteria isolated from raw foods including milk [33,40,41], bean sprouts [42], and fish, birds and mammal meat [40]. Extracts obtained from planktonic and biofilm cells of *H. alvei* contain HLs of different lengths (Table 1). At this point, we must reference the type of biosensor used to detect the AHLs produced by the different strains isolated from food. Strains of the species *Chromobacterium violaceum* are commonly used, mainly the CV026 strain. *C. violaceum* CV026 is a well-known AHL reporter strain, but this strain could not recognize long-acyl-chain homoserine lactones such as C10- and C14- HSLs. To do this, a new strain called VIR24 was developed, with the ability to detect long-acyl-chain HSL molecules not detected by CV026 [43]. Other species used to detect these molecules are genetically modified strains of *Agrobacterium tumefaciens*, *E. coli* and *Pseudomonas putida* [41]. Some HL molecules detected by CV biosensors are not detected by *Agrobacterium* or *E. coli* and vice versa. Hence the importance of using several biosensors to detect AHLs in different types of samples. Subsequently, chromatographic, or proteomic methods must be used to verify the type of molecule produced after comparison with known standards that can be obtained at a low price from different chemical reagent companies. N-Acyl-L-homoserine-lactone-producing *H. alvei* strains are frequently encountered in spoiling foods of protein origin. 

**Table 1:** Molecules produced by *H. alvei* strains related to food products.

Strain	Strain Source	Molecule	Biosensors Used	Characterization Method	Reference
068	Cooled raw milk	3-oxo-C6-HSL, 3-oxo-C8-HSL, C6-HLS	*C. violaceum CV026* *E. coli* MT102 *A. tumefaciens* A136 *A. tumefaciens* NTL4 *P. putida* F117	TLC, LC/MS	[41]
059 071	Cooled raw milk	3-oxo-C6-HSL, 3-oxo-C8-HSL, C6-HLS, C8-HLS	*C. violaceum* CV026 *A. tumefaciens* WCF47 *E. coli* Psb403	^2^ TLC, LC/MS	[33,41]
FB1	Vacuum-packed fish paste	3-oxo-C6-HSL, 3-oxo-C8-HSL	*C. violaceum* CV026	^1^ LC/MS	[44]
H4	Sea cucumber	C6-HSL, 3-oxo-C8-HSL, C4-HSL	CV026 and *A. tumefaciens* KYC55	TLC, LC/MS	[45]
718	Vacuum-packed meat	OHHL	*C. violaceum* CV026 *A. tumefaciens* pZLR4	TLC, LC/MS	[46]
Ha-01	Spoiled turbot	C6-HSL, C8-HSL	*C. violaceum* CV026	^3^ GC/MS	[47]

^1^ LC/MS: liquid chromatography/mass spectrometry; ^2^ Thin Layer Chromatography; ^3^ GC/MS: gas chromatography/mass spectrometry. C6-HSL (N-hexanoyl-L-homoserine lactone); 3-oxo-C8-HSL (N-(3-oxo-octanoyl)-L-homoserine lactone); C4-HSL (N-butyryl-L-homoserine lactone); OHHL (N-(3-oxo-hexanoyl) homoserine lactone).

*H. alvei* shares QS virulence factors with other pathogens of related enterobacterial species, therefore, it is not surprising that its adhesion properties to both inanimate surfaces and eukaryotic cells were studied, although not in depth. By presenting a group behavior, *H. alvei* is able to form biofilms on very diverse substrates and under different environmental conditions [48,49]. For example, in vacuum packaging and modified atmosphere packaging foods. Many strains of *H. alvei* are also able to grow at temperatures as low as 2.6 °C, making them a potential contaminant of refrigerated food [50]. However, the relationship between quorum sensing and biofilm production at refrigeration temperatures has not been studied in this bacterium. An interesting aspect of biofilm production in this species is the synthesis of cellulose. Some bacteria form biofilms by biogenesis of intracellular bis-(3′-5′) cyclic dimeric-GMP (c-di-GMP) which inhibits motility and induces secretion of biofilm-promoting adherence factors. c-di-GMP production has not been studied in this species but a set of genes—related to bcsABCD operon involved in the production of cellulose—during the formation of biofilms in this species was identified, and have their counterparts in other species of enterobacteria such as *E. coli* or *Salmonella* spp. [48]. Biosynthesis of cellulose-containing biofilms occurs in a variety of *Proteobacteria* that inhabit diverse ecological niches. Therefore, it is necessary to advance knowledge on the production of this compound not only in *H. alvei*, but also in other bacteria, since the control of biofilms represents one of the most persistent challenges to the food industry in which microbial communities of different foodborne pathogens are problematic. Quorum-quenching (QQ) mechanisms have recently been proposed as a strategy for controlling biofilm formation by interfering with this signaling system in food-borne bacteria [51,52,53,54,55]. In an interesting study, using a strain of *H. alvei* isolated from meat [56], Bruhn and coworkers demonstrated a cross-activity of quorum-sensing molecules produced by *H. alvei* on other species of bacteria—such as *Serratia proteomaculans*—that are isolated with some frequency from contaminated food, indicating that this species—and possibly other enterobacteria producing quorum-sensing molecules—participate in the spoilage of food products through interspecific communication and biofilm formation [46,57].

## 4. Biogenic Amines Produced by *H. alvei*

Biogenic amines (BAs) are organic nitrogenous compounds of low molecular weight formed mostly by decarboxylation of free amino acids—histidine, ornithine, lysine and tyrosine—mediated by amino acid decarboxylase enzymes. These biogenic amines are found in a wide range of foods including vegetables, milk products and meat, and also in some alcoholic beverages [58,59,60,61]. Gene sequences for lysine and ornithine decarboxylase were identified in the genome of a least one sequenced *H. alvei* strain [62].

The presence of biogenic amines in seafood is of interest because high levels of dietary biogenic amines can be toxic for consumers. High amounts of BAs in foods may cause gastric, cardiac, and intestinal problems, and also migraine and allergic responses in some individuals, as well. BAs are also of interest since they are a good index of food spoilage. Some common BAs found in spoiled foods are putrescine, cadaverine, spermidine, agmatine, tyramine and histamine [63,64,65]. 

Histamine is regarded as the main agent for scombrotoxic fish poisoning. The term “scombroid poisoning” was originally used to describe the illness, as this type of food poisoning was often associated with fish belonging to the family *Scombridae* (i.e., tuna). A correlation was shown between the concentration of histamine, cadaverine and putrescine and the sensory scores of sliced cold-smoked salmon [66]. Many Gram-negative and Gram-positive bacteria isolated from spoiled meat and meat products produce BAs under different conditions [67]. The most frequent Gram-negative bacterial species isolated from these products are members of the family *Enterobacteriaceae* [64,68,69]. Different articles have reported the production of these compounds by strains of *H. alvei*. In some cases, the number of BAs produced is high and similar to the number of BAs produced by strains of *E. coli*, *Morganella*, *Proteus*, *Citrobacter*, *Serratia* or *Providentia.*

In the repertoire of molecules of this type produced by *H. alvei* we find tryptamine, phenylethylamine, putrescine, cadaverine, histamine, tyramine, spermine and spermidine, although the concentration of BAs produced varies greatly for each particular strain [66,70,71,72,73,74,75]. As the biosynthesis of BA in foods requires the availability of key free amino acids, the good performance of *H. alvei* in cheese favors said biosynthesis. Monitoring the presence of these compounds produced by *H. alvei* and other bacteria during food processing and storage involves not only their detection but also a better understanding of the mechanisms when producing them. That is why more in vitro studies are needed to know the ability of *H. alvei* to produce these molecules in different food matrices and in the presence or absence of competing bacteria or chemical inhibitors. An example is the set of experiments performed by Dugat-Bony and coworkers with soft-ripened cheese, where the population of *H. alvei* was reduced with high content of NaCl, and concomitantly, the production of BA was also reduced [76].

## 5. Antagonistic Activity

The presence of genes for the production of bacteriocins was also discovered after an analysis of different genomes and plasmids of *H. alvei* [5,77]. Bacteriocins are molecules synthesized by several Gram-positive and Gram-negative bacteria with antimicrobial properties, even at low concentrations, provoking the inhibition of bacterial growth or directly killing the bacteria. Different researchers have found strains of *H. alvei* that have antagonistic activity against other bacteria for both clinical and food safety interests (Table 2). The antagonistic activity was demonstrated primarily by plate inhibition assays, directly confronting the strain of *H. alvei* that produces the inhibitor compound against the target strain. For example, strains of *H. alvei* with the capacity to produce antimicrobial compounds, such as bacteriocins, thiopeptides, and beta-lactones have recently been isolated [77,78]. Thiopeptide antibiotics are modified sulfur-rich peptides with a central nitrogen-containing six-membered ring that can be decorated with several azoles, such as thiazoles, and oxazoles with great activity against Gram-positive bacteria [79,80]. Beta-lactones were isolated from a variety of bacterial and fungal species which resemble the biologically highly active β-lactames [81]. Structurally, β-lactones are four-membered heterocycles with high ring-strain, electrophilicity and reactivity [82].

**Table 2:** Antagonistic activity of *H. alvei* against pathogens.

Source of H. *alvei*	Target Pathogen	Outcome	Inhibitor/Factor	Reference
Intestinal microbiota of humans and mammals	H. *alvei*	Killing of target strains	Alveicins A and B (pore forming bacteriocins)	[77]
Intestinal microbiota of rainbow trout	A. *hydrophila, L. monocytogenes, S. enterica, E. coli*	Growth inhibition	Putative bacteriocins	[78]
Meat	*Salmonella* Enteritidis	Inhibition of *Salmonella* biofilm Reduction of planktonic cell metabolism	Cell-free culture supernatant, non-enzymatic molecule	[83]
Honey bee gut	*Bacillus* sp.	Antagonistic activity	unknown	[5,84]
Cheese	^1^ STEC	Reduction in growth kinetics	unknown	[85]
Cheese	*E. coli* O26:H11	Reduction in growth kinetics	unknown	[86]
ATCC® 9760™	*E. coli* O157:H7	Reduction in growth kinetics	unknown	[87]
Cheese	*S. enterica* and *E. coli*	Killing of target strains	unknown	[88]
Cheese	*Staphylococcus aureus*	Killing of target strain and reduction of enterotoxin production	unknown	[89]
Raw ground pork	*Yersinia enterocolitica*	Growth inhibition	unknown	[90]

^1^ STEC: Shigatoxin-producing *Escherichia coli*.

Additionally, in the work of Awolope and coworkers [4], interesting siderophores were identified in the genome of an H. alveus strain isolated from the rhizosphere of a garden plant, which could be used as Trojan horses to facilitate the action of some antibiotics against resistant bacteria [91,92,93]. Interestingly, some *H. alvei* strains isolated from the gut microbiota of rainbow trout *Oncorhynchus mykiss* showed antagonistic activity to both Gram-negative pathogens (*Aeromonas hydrophila*, *Salmonella enterica*, and *Escherichia coli*) and Gram-positive pathogens (*Listeria monocytogenes*). Although the exact nature of the compounds involved in these effects was not fully characterized, the authors opt for some type of compound of a protein nature, possibly bacteriocins [78]. A clear example of the presence of these compounds with activity against strains of the same species or against other species is that of alveicins. These bacteriocins, similar to colicins produced by strains of *E. coli*, appear to be also encoded in plasmids [77,94,95]. The toxic action of these bacteriocins is derived from their ability to form a voltage-gated channel which causes depolarization of the membrane of sensitive bacterial cells. The role of *H. alvei* in the reduction of biofilms of other bacteria was also shown, including planktonic cells of important human pathogens present in food [83]. 

Fukushima and Gomyoda tested the antagonistic effect of *H. alvei* against *Yersinia enterocolitica* serotype O3 in raw ground pork. *H. alvei* was able to inhibit the growth of the *Y. enterocolitica* in this food matrix both at room temperature and at refrigeration temperature, but the exact mechanism of inhibition was not determined [90]. In one of the few studies in which strains of *H. alvei* were compared with strains of Gram-positive toxin-producing bacteria, an inhibitory effect of *Hafnia* was also demonstrated. The researchers showed that *H. alvei* reduces the number of *Staphylococcus aureus* in raw milk during the co-culture of both species [89]. Furthermore, this reduction was shown to also inhibit toxin production by *S. aureus*. The researchers found that milk acidification did not influence the growth of *S. aureus* or toxin production, so the most likely hypothesis for the inhibition was the production of some type of inhibitory compound by *H. alvei*. These data also demonstrated the ability of *H. alvei* to reduce the number of competing Gram-positive bacteria in dairy products. Another study conducted in Turkish meat products evidenced a high presence of *H. alvei*, which could be competing with *E. coli* O157:H7, isolated in low numbers; however, the assays were only observational, and no causality could be established to explain the low presence of *E. coli* in these samples or their competition with other bacteria such as *Citrobacter* or other non-O157:H7 strains of *E. coli* [96]. In some cases, direct competition assays between strains have not been able to determine an inhibitory effect, which does occur when competing strains are inoculated into a food matrix. This indicates that the production of certain bactericidal or bacteriostatic compounds is determined by the type of matrix or by the special nutrient utilization characteristics of competing species. For example, if competing species share patterns of carbon use, this could lead to them competing within the food matrix. Further characterization of these antibacterial products of *H. alvei* is essential for potential use in real conditions.

Sometimes, *H. alvei* is the bacterium that ends up displaced by a competitor. For example, strains of *Proteus vulgaris* reduce the number of *H. alvei* present in some types of cheese [97], and *Lactobacillus* spp. during the fermentation of table olives [98] or in vacuum-packaged steaks [99]. This last study again demonstrated the importance of both the growth temperature of the bacterium and the pH in the production of volatile compounds. Being a predominant bacterium in dairy products, there are not many species that can effectively compete against *H. alvei*, which in some cases would be desirable. To reduce the number of *H. alvei* in food, different strategies were used, such as the addition of dihydrocoumarin, a compound derived from coumarin, a known QS inhibitor that is used as an additive in food. In an inhibition study, dihydrocoumarin was shown to be able to reduce swimming motility and the biofilm formed by a strain of *H. alvei* isolated from ready-to-eat sea cucumber [100]. The inhibitory effect seems to be related to the action of this compound on the quorum-sensing molecules produced by *H. alvei*. Finally, furanones also affect biofilm formation in *H. alvei*, probably interfering with its QS communication system [33].

## 6. Other Compounds Produced by *H. alvei*

In addition to QS molecules, BAs, and antimicrobial compounds, we want to highlight that *H. alvei* produces three other types of molecules, which, although they are less studied, deserve to be commented on here: volatile compounds, molecules involved in the regulation of metabolism in mammals, and compounds of biotechnological interest. 

*H. alvei* is also able—as are several other bacterial species—to produce or induce the production of several volatile compounds in pure culture-inoculated beef samples stored aerobically at low temperatures. Among them, we can find alcohols such as 2-methylbutanol or 2,3-butanediol up to diacetyl, acetoin, dimethyl sulfide or methanethiol [101]. Of note, the production of these compounds is greatly influenced by the pH. *H. alvei* is able to generate volatile compounds, probably due to its capacity to transform the amino acids methionine and cysteine into aromatic compounds via transamination or deamination.

The growth of *H. alvei* also seems to favor the production of some volatile compounds by autochthonous populations. In a study using an experimental smear of soft cheese, the effect of *H. alvei* inoculation on the production of volatile compounds by the indigenous microbial communities of this type of cheese was demonstrated [102]. The compounds that were mostly induced after inoculation with *H. alvei* were methanethiol, methythioacetate, dimethyl disulfide and dimethyl trisulfide, which can be responsible for the quality and uniqueness of a variety of cheeses. 

As we mentioned earlier, *H. alvei* is frequently detected in traditional cheeses [103,104,105,106,107,108,109]. In a study in which 17 species of bacteria were identified in soft raw ewe’s milk cheeses, it was determined that *H. paralvei* and *H. alvei* were predominant species, which gives us an idea of the ubiquity of these related species in cheeses of different origin and geographic location [110].

The variety of molecules produced by *H. alvei* when it has favorable conditions to grow into cheese was made clear in a study carried out with the reference strain ATCC^®^ 51815^TM^ —isolated from milk—which was used to modify some organoleptic properties of ewe milk cheese. Researchers separately used the extracellular products of this species—and also from other bacteria and yeasts—to take advantage of the enzymatic activities and accelerate or condition cheese ripening without modifying the main compositional features of the cheese. The main modification made by the extracellular products of *H. alvei* in this work was the increase in the synthesis of sulfur compounds such as methanethiol, dimethyl-trisulfide, 2.4-dithiapentane, 3-methyl-thio-1-propene, s-methyl thioacetate and 1-methyl-thiopropane, and the increasing reduction of some esters such as methyl butanoate, ethyl acetate, propyl butanoate, 1-methyl-propyl butanoate, 2-methyl-butyl acetate, and 3-methyl-butyl acetate [111]. The same authors performed a similar study using the extracellular products of *H. alvei* to modify the sensory and biochemical characteristics of sourdough bread [112]. In a similar work, Spanish researchers evaluated the production of volatile compounds by four strains of *H. alvei* isolated from dairy products [113]. The production of compounds in the internal part of these cheeses evolved over time from 24h to 8 days, with an increase in the levels of compounds such as 2-methyl propanal, 2,3-butanedione, 3-methyl butanol, and dimethyl sulfide. This last was observed inside cheeses, although in many cases, these levels were comparable to those induced by strains of other species of enterobacteria such as *Serratia liquefaciens*, *Enterobacter sakazakii*, *E. cloacae*, or *E. coli*.

*H. alvei* could also be interesting in regulating metabolism when introduced as a probiotic. Based on the production of the caseinolytic protease B (Clpb) protein, identified as a key anorexigenic peptide involved in the regulation of appetite, researchers have used the strain HA4597^TM^ as a probiotic in a mouse model of hyperphagic obesity. The results demonstrate some beneficial anti-obesity and metabolic effects induced by the bacterium, which may represent a strategy for long-term body weight management [28,29]. When authorizing probiotics for human health, the strains must meet special characteristics, by which it would be interesting to know the complete genome of this patented strain, as well as the presence or absence of virulence determinants or resistance genes. Furthermore, it would be interesting to compare this genome with other strains of the same or different species, in order to select the best probiotic candidates.

In another interesting study, German researchers showed that metabolites generated during the growth of *H. alvei* improved the inflammatory response of endothelial cells by regulating the levels of their adhesion molecules and reducing the production of proinflammatory cytokines such as IL-6 and IL-8 [114]. These findings could have relevance for the understanding, treatment or reduction of symptoms or low-grade inflammation in some inflammatory diseases.

The induction of pro- or anti-inflammatory cytokines by *H. alvei* is a little explored field. For example, it was shown that the lipopolysaccharide (LPS) from certain *H. alvei* strains induces high levels of the cytokine Interleukin-10 (IL-10) in dendritic cells [115]. IL-10 is an anti-inflammatory cytokine that modulates innate and adaptive immunity. The induction or repression of cytokines by some *H. alvei* strains may help us to improve our understanding of some aspects of host–microbe interactions both with commensal, probiotic or pathogenic strains.

To conclude this review, we would like to cite *H. alvei* as a possible biofactory. 1,3-Propanediol is synthesized during glycerol fermentation and has a number of industrial applications. As 1,3-propanediol can be produced through a biotechnological process by bacteria, *H. alvei* was postulated as an alternative biocatalyst for the production of this compound [116]. The strain AD27 used in this study was isolated from pickled vegetables, and its rapid growth and easy genetic manipulation make it a bacterium with good prospects in the biotech industry [117,118]. 

## 7. Concluding Remarks

With the advent of genome sequence information, we came across a lot of information which then causes the problem of being able to apply this information to biological questions. At the moment, this problem does not yet occur with *H. alvei*, since unfortunately there are only half a dozen complete genomes available, and only one of them belongs to a strain isolated from food. Information from a representative number of genomes could help us identify molecules produced by this species in different fields, such as bacteriocins, the regulation of enzymatic activities by quorum sensing, the production of secondary metabolites beneficial or harmful to food, or the potential use of the antimicrobial compounds produced by *H. alvei*. Although some of these molecules have been partially identified and characterized, there is a long way to go until they can be used in real conditions, such as in food preservation. 

In the event that the molecules produced by *H. alvei* are harmful, a strategy to favor its inhibition within food matrices would be the use of compounds that block quorum-sensing signaling pathways in this bacterium, for example, using products that perform quorum quenching. On the contrary, some of the products of its metabolism seem to have an interest both in the medicine and in the food industry, either because they directly inhibit the growth of other microorganisms, or because they are susceptible to biotechnological modification that makes them applicable in real life. We must continue to advance in the characterization of the antimicrobial products secreted by *H. alvei*, since these compounds act by inhibiting or delaying the growth of other species of important Gram-negative and Gram-positive bacteria, which can contribute positively to extending the shelf life of cheeses and other dairy products.

Finally, as *H. alvei* strains have very diverse origins, the sequencing of new genomes will add new molecules to the list of potentially interesting compounds produced by this bacterium, susceptible to modification, or that can be directly applied to food.

## References

[1] Adeolu M., Alnajar S., Naushad S., Gupta R.S. (2016). Genome-Based Phylogeny and Taxonomy of the “Enterobacteriales”: Proposal for *Enterobacterales* Ord. Nov. Divided into the Families *Enterobacteriaceae*, *Erwiniaceae* Fam. Nov., *Pectobacteriaceae* Fam. Nov., *Yersiniaceae* Fam. Nov., *Hafniaceae* Fam. Nov., *Morganellaceae* Fam. Nov., and *Budviciaceae* Fam. Nov. Int. J. Syst. Evol. Microbiol..

[2] Padilla D., Remuzgo-Martínez S., Acosta F., Ramos-Vivas J. (2013). *Hafnia alvei* and *Hafnia paralvei*. Taxonomy Defined but Still Far from Virulence and Pathogenicity. Vet. Microbiol..

[3] Rodríguez L.A., Vivas J., Gallardo C.S., Acosta F., Barbeyto L., Real F. (1999). Identification of *Hafnia alvei* with the MicroScan WalkAway System. J. Clin. Microbiol..

[4] Awolope O.K., O’Driscoll N.H., Di Salvo A., Lamb A.J. (2021). The Complete Genome Sequence of *Hafnia alvei* A23BA; a Potential Antibiotic-Producing Rhizobacterium. BMC Res. Notes.

[5] Tian B., Moran N.A. (2016). Genome Sequence of *Hafnia alvei* Bta3_1, a Bacterium with Antimicrobial Properties Isolated from Honey Bee Gut. Genome Announc..

[6] Lang H., Duan H., Wang J., Zhang W., Guo J., Zhang X., Hu X., Zheng H. (2022). Specific Strains of Honeybee Gut *Lactobacillus* Stimulate Host Immune System to Protect against Pathogenic *Hafnia alvei*. Microbiol. Spectr..

[7] Ionescu M.I., Neagoe D.Ș., Crăciun A.M., Moldovan O.T. (2022). The Gram-Negative Bacilli Isolated from Caves-*Sphingomonas paucimobilis* and *Hafnia alvei* and a Review of Their Involvement in Human Infections. Int. J. Environ. Res. Public Health.

[8] Abbott S.L., Moler S., Green N., Tran R.K., Wainwright K., Janda J.M. (2011). Clinical and Laboratory Diagnostic Characteristics and Cytotoxigenic Potential of *Hafnia alvei* and *Hafnia paralvei* Strains. J. Clin. Microbiol..

[9] Huys G., Cnockaert M., Abbott S.L., Janda J.M., Vandamme P. (2010). *Hafnia paralvei* Sp. Nov., Formerly Known as *Hafnia alvei* Hybridization Group 2. Int. J. Syst. Evol. Microbiol..

[10] Wang T.K.F., Yam W.-C., Yuen K.-Y., Wong S.S.Y. (2006). Misidentification of a Mucoid Strain of *Salmonella enterica serotype choleraesuis* as *Hafnia alvei* by the Vitek GNI+ Card System. J. Clin. Microbiol..

[11] Wright W.F., Utz J.L., Bruckhart C., Baghli S., Janda J.M. (2019). *Yokenella regensburgei* Necrotizing Fasciitis in an Immunocompromised Host. J. Infect. Chemother..

[12] Koivula T.T., Juvonen R., Haikara A., Suihko M.-L. (2006). Characterization of the Brewery Spoilage Bacterium *Obesumbacterium proteus* by Automated Ribotyping and Development of PCR Methods for Its Biotype 1. J. Appl. Microbiol..

[13] Vithanage N.R., Yeager T.R., Jadhav S.R., Palombo E.A., Datta N. (2014). Comparison of Identification Systems for Psychrotrophic Bacteria Isolated from Raw Bovine Milk. Int. J. Food Microbiol..

[14] Höll L., Behr J., Vogel R.F. (2016). Identification and Growth Dynamics of Meat Spoilage Microorganisms in Modified Atmosphere Packaged Poultry Meat by MALDI-TOF MS. Food Microbiol..

[15] Padilla D., Acosta F., Bravo J., Grasso V., Real F., Vivas J. (2008). Invasion and Intracellular Survival of *Hafnia alvei* Strains in Human Epithelial Cells. J. Appl. Microbiol..

[16] Ramos-Vivas J. (2020). Microbiology of *Hafnia alvei*. Enferm. Infecc. Microbiol Clin..

[17] Padilla D., Acosta F., García J.A., Real F., Vivas J.R. (2009). Temperature Influences the Expression of Fimbriae and Flagella in *Hafnia alvei* Strains: An Immunofluorescence Study. Arch. Microbiol..

[18] L D., Acosta F., Ramos-Vivas J., Grasso V., Bravo J., El Aamri F., Real F. (2015). The Pathogen *Hafnia alvei* in Veterinary Medicine: A Review. J. Appl. Anim. Res..

[19] Lindberg A.M., Ljungh A., Ahrné S., Löfdahl S., Molin G. (1998). *Enterobacteriaceae* Found in High Numbers in Fish, Minced Meat and Pasteurised Milk or Cream and the Presence of Toxin Encoding Genes. Int. J. Food Microbiol..

[20] Anjum M., Madsen J.S., Nesme J., Jana B., Wiese M., Jasinskytė D., Nielsen D.S., Sørensen S.J., Dalsgaard A., Moodley A. (2019). Fate of CMY-2-Encoding Plasmids Introduced into the Human Fecal Microbiota by Exogenous *Escherichia coli*. Antimicrob. Agents Chemother..

[21] Nadjar D., Rouveau M., Verdet C., Donay L., Herrmann J., Lagrange P.H., Philippon A., Arlet G. (2000). Outbreak of *Klebsiella Pneumoniae* Producing Transferable AmpC-Type Beta-Lactamase (ACC-1) Originating from *Hafnia alvei*. FEMS Microbiol. Lett..

[22] Agga G.E., Silva P.J., Martin R.S. (2021). Detection of Extended-Spectrum Beta-Lactamase-Producing and Carbapenem-Resistant Bacteria from Mink Feces and Feed in the United States. Foodborne Pathog. Dis..

[23] Jayol A., Saly M., Nordmann P., Ménard A., Poirel L., Dubois V. (2017). *Hafnia*, an Enterobacterial Genus Naturally Resistant to Colistin Revealed by Three Susceptibility Testing Methods. J. Antimicrob. Chemother..

[24] Zurfluh K., Stephan R., Widmer A., Poirel L., Nordmann P., Nüesch H.-J., Hächler H., Nüesch-Inderbinen M. (2017). Screening for Fecal Carriage of MCR-Producing *Enterobacteriaceae* in Healthy Humans and Primary Care Patients. Antimicrob. Resist. Infect. Control.

[25] Crandall C., Abbott S.L., Zhao Y.Q., Probert W., Janda J.M. (2006). Isolation of Toxigenic *Hafnia alvei* from a Probable Case of Hemolytic Uremic Syndrome. Infection.

[26] Patterson J.T., Gibbs P.A. (1978). Sources and Properties of Some Organisms Isolated in Two Abattoirs. Meat Sci..

[27] Bourdichon F., Casaregola S., Farrokh C., Frisvad J.C., Gerds M.L., Hammes W.P., Harnett J., Huys G., Laulund S., Ouwehand A. (2012). Food Fermentations: Microorganisms with Technological Beneficial Use. Int. J. Food Microbiol..

[28] Legrand R., Lucas N., Dominique M., Azhar S., Deroissart C., Le Solliec M.-A., Rondeaux J., Nobis S., Guérin C., Léon F. (2020). Commensal *Hafnia alvei* Strain Reduces Food Intake and Fat Mass in Obese Mice-a New Potential Probiotic for Appetite and Body Weight Management. Int. J. Obes..

[29] Lucas N., Legrand R., Deroissart C., Dominique M., Azhar S., Le Solliec M.-A., Léon F., do Rego J.-C., Déchelotte P., Fetissov S.O. (2019). *Hafnia alvei* HA4597 Strain Reduces Food Intake and Body Weight Gain and Improves Body Composition, Glucose, and Lipid Metabolism in a Mouse Model of Hyperphagic Obesity. Microorganisms.

[30] Lázaro-Díez M., Redondo-Salvo S., Arboleya-Agudo A., Ocejo-Vinyals J.G., Chapartegui-González I., Ocampo-Sosa A.A., Acosta F., Martínez-Martínez L., Ramos-Vivas J. (2016). Whole-Genome Sequence of *Hafnia alvei* HUMV-5920, a Human Isolate. Genome Announc..

[31] Li X., Zhang G., Zhu Y., Bi J., Hao H., Hou H. (2019). Effect of the LuxI/R Gene on AHL-Signaling Molecules and QS Regulatory Mechanism in *Hafnia alvei* H4. AMB Express.

[32] Lazar V., Holban A.M., Curutiu C., Chifiriuc M.C. (2021). Modulation of Quorum Sensing and Biofilms in Less Investigated Gram-Negative ESKAPE Pathogens. Front. Microbiol..

[33] Viana E.S., Campos M.E.M., Ponce A.R., Mantovani H.C., Vanetti M.C.D. (2009). Biofilm Formation and Acyl Homoserine Lactone Production in *Hafnia alvei* Isolated from Raw Milk. Biol. Res..

[34] Remuzgo-Martínez S., Lázaro-Díez M., Mayer C., Aranzamendi-Zaldumbide M., Padilla D., Calvo J., Marco F., Martínez-Martínez L., Icardo J.M., Otero A. (2015). Biofilm Formation and Quorum-Sensing-Molecule Production by Clinical Isolates of *Serratia liquefaciens*. Appl. Environ. Microbiol..

[35] Vivas J., Razquin B.E., López-Fierro P., Naharro G., Villena A. (2004). Correlation between Production of Acyl Homoserine Lactones and Proteases in an *Aeromonas hydrophila* AroA Live Vaccine. Vet. Microbiol..

[36] Yan J., Yang Z., Xie J. (2022). Comparative Transcriptome Analysis of *Shewanella putrefaciens* WS13 Biofilms Under Cold Stress. Front. Cell. Infect. Microbiol..

[37] Popoff M.R., Brüggemann H. (2022). Regulatory Networks Controlling Neurotoxin Synthesis in *Clostridium botulinum* and *Clostridium tetani*. Toxins.

[38] Li T., Yang B., Li X., Li J., Zhao G., Kan J. (2018). Quorum Sensing System and Influence on Food Spoilage in *Pseudomonas fluorescens* from Turbot. J. Food Sci. Technol..

[39] Smith J.L., Fratamico P.M., Yan X. (2011). Eavesdropping by Bacteria: The Role of SdiA in *Escherichia coli* and *Salmonella enterica serovar typhimurium* Quorum Sensing. Foodborne Pathog. Dis..

[40] Liu M., Gray J.M., Griffiths M.W. (2006). Occurrence of Proteolytic Activity and N-Acyl-Homoserine Lactone Signals in the Spoilage of Aerobically Chill-Stored Proteinaceous Raw Foods. J. Food Prot..

[41] Martins M.L., Pinto U.M., Riedel K., Vanetti M.C.D. (2018). Quorum Sensing and Spoilage Potential of Psychrotrophic Enterobacteriaceae Isolated from Milk. Biomed. Res. Int..

[42] Rasch M., Andersen J.B., Nielsen K.F., Flodgaard L.R., Christensen H., Givskov M., Gram L. (2005). Involvement of Bacterial Quorum-Sensing Signals in Spoilage of Bean Sprouts. Appl. Environ. Microbiol..

[43] Someya N., Morohoshi T., Okano N., Otsu E., Usuki K., Sayama M., Sekiguchi H., Ikeda T., Ishida S. (2009). Distribution of N-Acylhomoserine Lactone-Producing *Fluorescent pseudomonads* in the Phyllosphere and Rhizosphere of Potato (*Solanum tuberosum* L.). Microbes Environ..

[44] Tan J.-Y., Yin W.-F., Chan K.-G. (2014). Quorum Sensing Activity of *Hafnia alvei* Isolated from Packed Food. Sensors.

[45] Hou H.-M., Zhu Y.-L., Wang J.-Y., Jiang F., Qu W.-Y., Zhang G.-L., Hao H.-S. (2017). Characteristics of N-Acylhomoserine Lactones Produced by *Hafnia alvei* H4 Isolated from Spoiled Instant Sea Cucumber. Sensors.

[46] Bruhn J.B., Christensen A.B., Flodgaard L.R., Nielsen K.F., Larsen T.O., Givskov M., Gram L. (2004). Presence of Acylated Homoserine Lactones (AHLs) and AHL-Producing Bacteria in Meat and Potential Role of AHL in Spoilage of Meat. Appl. Environ. Microbiol..

[47] Li T., Mei Y., He B., Sun X., Li J. (2018). Reducing Quorum Sensing-Mediated Virulence Factor Expression and Biofilm Formation in *Hafnia alvei* by Using the Potential Quorum Sensing Inhibitor L-Carvone. Front. Microbiol..

[48] Chapartegui-González I., Lázaro-Díez M., Redondo-Salvo S., Amaro-Prellezo E., Esteban-Rodríguez E. (2016). Biofilm Formation in *Hafnia alvei* HUMV-5920, a Human Isolate. AIMS Microbiol..

[49] Vivas J., Padilla D., Real F., Bravo J., Grasso V., Acosta F. (2008). Influence of Environmental Conditions on Biofilm Formation by *Hafnia alvei* Strains. Vet. Microbiol..

[50] Ridell J., Korkeala H. (1997). Minimum Growth Temperatures of *Hafnia alvei* and Other *Enterobacteriaceae* Isolated from Refrigerated Meat Determined with a Temperature Gradient Incubator. Int. J. Food Microbiol..

[51] Mayer C., Muras A., Parga A., Romero M., Rumbo-Feal S., Poza M., Ramos-Vivas J., Otero A. (2020). Quorum Sensing as a Target for Controlling Surface Associated Motility and Biofilm Formation in *Acinetobacter baumannii* ATCC® 17978TM. Front. Microbiol..

[52] Cabo M.L., Rodríguez A., Herrera J.R. (2022). Exploring Communication Signals inside the Microbial Community of a *Listeria monocytogenes*-Carrying Biofilm Contamination Site. Int. J. Food Microbiol..

[53] Prazdnova E.V., Gorovtsov A.V., Vasilchenko N.G., Kulikov M.P., Statsenko V.N., Bogdanova A.A., Refeld A.G., Brislavskiy Y.A., Chistyakov V.A., Chikindas M.L. (2022). Quorum-Sensing Inhibition by Gram-Positive Bacteria. Microorganisms.

[54] Shen Y., Cui F., Wang D., Li T., Li J. (2021). Quorum Quenching Enzyme (PF-1240) Capable to Degrade AHLs as a Candidate for Inhibiting Quorum Sensing in Food Spoilage Bacterium *Hafnia alvei*. Foods.

[55] Wang D., Chen H., Li J., Li T., Ren L., Liu J., Shen Y. (2022). Screening and Validation of Quorum Quenching Enzyme PF2571 from *Pseudomonas fluorescens* Strain PF08 to Inhibit the Spoilage of Red Sea Bream Filets. Int. J. Food Microbiol..

[56] Borch E., Kant-Muermans M.L., Blixt Y. (1996). Bacterial Spoilage of Meat and Cured Meat Products. Int. J. Food Microbiol..

[57] Smith J.-L., Fratamico P.-M., Novak J.-S. (2004). Quorum sensing: A primer for food microbiologists. J. Food Prot..

[58] Schirone M., Esposito L., D’Onofrio F., Visciano P., Martuscelli M., Mastrocola D., Paparella A. (2022). Biogenic Amines in Meat and Meat Products: A Review of the Science and Future Perspectives. Foods.

[59] Sivamaruthi B.S., Kesika P., Chaiyasut C. (2021). A Narrative Review on Biogenic Amines in Fermented Fish and Meat Products. J. Food Sci. Technol..

[60] Ruiz-Capillas C., Herrero A.M. (2019). Impact of Biogenic Amines on Food Quality and Safety. Foods.

[61] Moniente M., García-Gonzalo D., Ontañón I., Pagán R., Botello-Morte L. (2021). Histamine Accumulation in Dairy Products: Microbial Causes, Techniques for the Detection of Histamine-Producing Microbiota, and Potential Solutions. Compr. Rev. Food Sci. Food Saf..

[62] Tan J.-Y., Yin W.-F., Chan K.-G. (2014). Gene Clusters of *Hafnia alvei* Strain FB1 Important in Survival and Pathogenesis: A Draft Genome Perspective. Gut Pathog..

[63] Vasconcelos H., Coelho L.C.C., Matias A., Saraiva C., Jorge P.A.S., de Almeida J.M.M.M. (2021). Biosensors for Biogenic Amines: A Review. Biosensors.

[64] Visciano P., Schirone M., Paparella A. (2020). An Overview of Histamine and Other Biogenic Amines in Fish and Fish Products. Foods.

[65] Jørgensen L.V., Dalgaard P., Huss H.H. (2000). Multiple Compound Quality Index for Cold-Smoked Salmon (*Salmo salar*) Developed by Multivariate Regression of Biogenic Amines and PH. J. Agric. Food Chem..

[66] Jørgensen L.V., Huss H.H., Dalgaard P. (2000). The Effect of Biogenic Amine Production by Single Bacterial Cultures and Metabiosis on Cold-Smoked Salmon. J. Appl. Microbiol..

[67] Lee Y.-C., Kung H.-F., Wu C.-H., Hsu H.-M., Chen H.-C., Huang T.-C., Tsai Y.-H. (2016). Determination of Histamine in Milkfish Stick Implicated in Food-Borne Poisoning. J. Food Drug Anal..

[68] Durlu-Özkaya F., Ayhan K., Vural N. (2001). Biogenic Amines Produced by *Enterobacteriaceae* Isolated from Meat Products. Meat Sci..

[69] Lorenzo J.M., Cachaldora A., Fonseca S., Gómez M., Franco I., Carballo J. (2010). Production of Biogenic Amines “in vitro” in Relation to the Growth Phase by *Enterobacteriaceae* Species Isolated from Traditional Sausages. Meat Sci..

[70] Gullian Klanian M., Delgadillo Díaz M., Sánchez Solís M.J. (2018). Molecular Characterization of Histamine-Producing Psychrotrophic Bacteria Isolated from Red Octopus (*Octopus maya*) in Refrigerated Storage. High. Throughput.

[71] Lavizzari T., Breccia M., Bover-Cid S., Vidal-Carou M.C., Veciana-Nogués M.T. (2010). Histamine, Cadaverine, and Putrescine Produced in Vitro by *Enterobacteriaceae* and *Pseudomonadaceae* Isolated from Spinach. J. Food Prot..

[72] Kim S.H., Field K.G., Morrissey M.T., Price R.J., Wei C.I., An H. (2001). Source and Identification of Histamine-Producing Bacteria from Fresh and Temperature-Abused Albacore. J. Food Prot..

[73] Dainty R.H., Edwards R.A., Hibbard C.M., Ramantanis S.V. (1986). Bacterial Sources of Putrescine and Cadaverine in Chill Stored Vacuum-Packaged Beef. J. Appl. Bacteriol..

[74] Hwang C.-C., Tseng P.-H., Lee Y.-C., Kung H.-F., Huang C.-Y., Chen H.-C., Tsai Y.-H. (2019). Determination of Histamine in Japanese Spanish Mackerel (*Scomberomorus niphonius*) Meat Implicated in a Foodborne Poisoning. J. Food Prot..

[75] Bremer P.J., Osborne C.M., Kemp R.A., van Veghel P., Fletcher G.C. (1998). Thermal Death Times of *Hafnia alvei* Cells in a Model Suspension and in Artificially Contaminated Hot-Smoked Kahawai (Arripis Trutta). J. Food Prot..

[76] Dugat-Bony E., Sarthou A.-S., Perello M.-C., de Revel G., Bonnarme P., Helinck S. (2016). The Effect of Reduced Sodium Chloride Content on the Microbiological and Biochemical Properties of a Soft Surface-Ripened Cheese. J. Dairy Sci..

[77] Wertz J.E., Riley M.A. (2004). Chimeric Nature of Two Plasmids of *Hafnia alvei* Encoding the Bacteriocins Alveicins A and B. J. Bacteriol..

[78] Lobos O., Barrera A., Padilla C. (2017). Microorganisms of the Intestinal Microbiota of *Oncorhynchus mykiss* Produce Antagonistic Substances against Bacteria Contaminating Food and Causing Disease in Humans. Ital. J. Food Saf..

[79] Just-Baringo X., Albericio F., Álvarez M. (2014). Thiopeptide Antibiotics: Retrospective and Recent Advances. Mar. Drugs.

[80] Chan D.C.K., Burrows L.L. (2021). Thiopeptides: Antibiotics with Unique Chemical Structures and Diverse Biological Activities. J. Antibiot..

[81] Robinson S.L., Christenson J.K., Wackett L.P. (2019). Biosynthesis and Chemical Diversity of β-Lactone Natural Products. Nat. Prod. Rep..

[82] Schaffer J.E., Reck M.R., Prasad N.K., Wencewicz T.A. (2017). β-Lactone Formation during Product Release from a Nonribosomal Peptide Synthetase. Nat. Chem. Biol..

[83] Chorianopoulos N.G., Giaouris E.D., Kourkoutas Y., Nychas G.-J.E. (2010). Inhibition of the Early Stage of *Salmonella enterica serovar enteritidis* Biofilm Development on Stainless Steel by Cell-Free Supernatant of a *Hafnia alvei* Culture. Appl. Environ. Microbiol..

[84] Tian B., Fadhil N.H., Powell J.E., Kwong W.K., Moran N.A. (2012). Long-Term Exposure to Antibiotics Has Caused Accumulation of Resistance Determinants in the Gut Microbiota of Honeybees. mBio.

[85] Callon C., Arliguie C., Montel M.-C. (2016). Control of Shigatoxin-Producing *Escherichia coli* in Cheese by Dairy Bacterial Strains. Food Microbiol..

[86] Delbès-Paus C., Miszczycha S., Ganet S., Helinck S., Veisseire P., Pochet S., Thévenot D., Montel M.-C. (2013). Behavior of *Escherichia coli* O26:H11 in the Presence of *Hafnia Alvei* in a Model Cheese Ecosystem. Int. J. Food Microbiol..

[87] Duffy G., Whiting R.C., Sheridan J.J. (1999). The Effect of a Competitive Microflora, PH and Temperature on the Gworth Kinetics of *Escherichia coli* O157:H7. Food Microbiol..

[88] Damaceno H.F.B., de Freitas C.V., Marinho I.L., Cupertino T.R., de Oliveira Costa L.E., dos Santos Nascimento J. (2015). Antibiotic Resistance versus Antimicrobial Substances Production by Gram-Negative Foodborne Pathogens Isolated from Minas Frescal Cheese: Heads or Tails?. Foodborne Pathog. Dis..

[89] Aljasir S.F., D’Amico D.J. (2020). The Effect of Protective Cultures on *Staphylococcus aureus* Growth and Enterotoxin Production. Food Microbiol..

[90] Fukushima H., Gomyoda M. (1986). Inhibition of *Yersinia enterocolitica* Serotype O3 by Natural Microflora of Pork. Appl. Environ. Microbiol..

[91] Ribeiro M., Sousa C.A., Simões M. (2022). Harnessing Microbial Iron Chelators to Develop Innovative Therapeutic Agents. J. Adv. Res..

[92] Khasheii B., Mahmoodi P., Mohammadzadeh A. (2021). Siderophores: Importance in Bacterial Pathogenesis and Applications in Medicine and Industry. Microbiol. Res..

[93] Górska A., Sloderbach A., Marszałł M.P. (2014). Siderophore-Drug Complexes: Potential Medicinal Applications of the “Trojan Horse” Strategy. Trends Pharmacol. Sci..

[94] Cascales E., Buchanan S.K., Duché D., Kleanthous C., Lloubès R., Postle K., Riley M., Slatin S., Cavard D. (2007). Colicin Biology. Microbiol. Mol. Biol. Rev..

[95] Cramer W.A., Heymann J.B., Schendel S.L., Deriy B.N., Cohen F.S., Elkins P.A., Stauffacher C.V. (1995). Structure-Function of the Channel-Forming Colicins. Annu. Rev. Biophys. Biomol. Struct..

[96] Noveir M.R., Dogan H.B., Kadir Halkman A. (2000). A Note on *Escherichia coli* O157:H7 Serotype in Turkish Meat Products. Meat Sci..

[97] Deetae P., Mounier J., Bonnarme P., Spinnler H.E., Irlinger F., Helinck S. (2009). Effects of *Proteus vulgaris* Growth on the Establishment of a Cheese Microbial Community and on the Production of Volatile Aroma Compounds in a Model Cheese. J. Appl. Microbiol..

[98] De Angelis M., Campanella D., Cosmai L., Summo C., Rizzello C.G., Caponio F. (2015). Microbiota and Metabolome of Un-Started and Started Greek-Type Fermentation of *Bella di cerignola* Table Olives. Food Microbiol..

[99] Hanna M.O., Smith G.C., Hall L.C., Vanderzant C. (1979). Role of *Hafnia alvei* and a *Lactobacillus* Species in the Spoilage of Vacuum-Packaged Strip Loin Steaks. J. Food Prot..

[100] Hou H.M., Jiang F., Zhang G.L., Wang J.Y., Zhu Y.H., Liu X.Y. (2017). Inhibition of *Hafnia Alvei* H4 Biofilm Formation by the Food Additive Dihydrocoumarin. J. Food Prot..

[101] Dainty R.H., Edwards R.A., Hibbard C.M., Marnewick J.J. (1989). Volatile Compounds Associated with Microbial Growth on Normal and High PH Beef Stored at Chill Temperatures. J. Appl. Bacteriol..

[102] Irlinger F., Yung S.A.Y.I., Sarthou A.-S., Delbès-Paus C., Montel M.-C., Coton E., Coton M., Helinck S. (2012). Ecological and Aromatic Impact of Two Gram-Negative Bacteria (*Psychrobacter celer* and *Hafnia alvei*) Inoculated as Part of the Whole Microbial Community of an Experimental Smear Soft Cheese. Int. J. Food Microbiol..

[103] Abriouel H., Martín-Platero A., Maqueda M., Valdivia E., Martínez-Bueno M. (2008). Biodiversity of the Microbial Community in a Spanish Farmhouse Cheese as Revealed by Culture-Dependent and Culture-Independent Methods. Int. J. Food Microbiol..

[104] Mounier J., Monnet C., Jacques N., Antoinette A., Irlinger F. (2009). Assessment of the Microbial Diversity at the Surface of Livarot Cheese Using Culture-Dependent and Independent Approaches. Int. J. Food Microbiol..

[105] Chaves-López C., De Angelis M., Martuscelli M., Serio A., Paparella A., Suzzi G. (2006). Characterization of the *Enterobacteriaceae* Isolated from an Artisanal Italian Ewe’s Cheese (Pecorino Abruzzese). J. Appl. Microbiol.

[106] Gaya P., Medina M., Nuñez M. (1987). *Enterobacteriaceae*, *Coliforms*, *Faecal coliforms* and *Salmonellas* in Raw Ewes’ Milk. J. Appl. Bacteriol..

[107] Maifreni M., Frigo F., Bartolomeoli I., Innocente N., Biasutti M., Marino M. (2013). Identification of the Enterobacteriaceae in Montasio Cheese and Assessment of Their Amino Acid Decarboxylase Activity. J. Dairy Res..

[108] Tabla R., Gómez A., Simancas A., Rebollo J.E., Molina F., Roa I. (2018). Early Blowing in Raw Goats’ Milk Cheese: Gas Production Capacity of *Enterobacteriaceae* Species Present during Manufacturing and Ripening. J. Dairy Res..

[109] Freitas A.C., Pais C., Malcata F.X., Hogg T.A. (1996). Microbiological Characterization of Picante Da Beira Baixa Cheese. J. Food Prot..

[110] Merchán A.V., Ruiz-Moyano S., Hernández M.V., Martín A., Lorenzo M.J., Benito M.J. (2022). Characterization of Autochthonal *Hafnia* Spp. Strains Isolated from Spanish Soft Raw Ewe’s Milk PDO Cheeses to Be Used as Adjunct Culture. Int. J. Food Microbiol..

[111] Calasso M., Mancini L., Di Cagno R., Cardinali G., Gobbetti M. (2015). Microbial Cell-Free Extracts as Sources of Enzyme Activities to Be Used for Enhancement Flavor Development of Ewe Milk Cheese. J. Dairy Sci..

[112] Cavallo N., De Angelis M., Calasso M., Quinto M., Mentana A., Minervini F., Cappelle S., Gobbetti M. (2017). Microbial Cell-Free Extracts Affect the Biochemical Characteristics and Sensorial Quality of Sourdough Bread. Food Chem..

[113] Morales P., Feliu I., Fernández-García E., Nuñez M. (2004). Volatile Compounds Produced in Cheese by *Enterobacteriaceae* Strains of Dairy Origin. J. Food Prot..

[114] Kuntz S., Kunz C., Domann E., Würdemann N., Unger F., Römpp A., Rudloff S. (2016). Inhibition of Low-Grade Inflammation by Anthocyanins after Microbial Fermentation In Vitro. Nutrients.

[115] Wittmann A., Lamprinaki D., Bowles K.M., Katzenellenbogen E., Knirel Y.A., Whitfield C., Nishimura T., Matsumoto N., Yamamoto K., Iwakura Y. (2016). Dectin-2 Recognizes Mannosylated O-Antigens of Human Opportunistic Pathogens and Augments Lipopolysaccharide Activation of Myeloid Cells. J. Biol. Chem..

[116] Celińska E., Drożdżyńska A., Wita A., Juzwa W., Białas W., Czaczyk K., Grajek W. (2017). Group II Intron-Mediated Deletion of Lactate Dehydrogenase Gene in an Isolated 1,3-Propanediol Producer *Hafnia alvei* AD27. Acta Biochim. Pol..

[117] Drożdżyńska A., Pawlicka J., Kubiak P., Kośmider A., Pranke D., Olejnik-Schmidt A., Czaczyk K. (2014). Conversion of Glycerol to 1,3-Propanediol by *Citrobacter freundii* and *Hafnia alvei*—Newly Isolated Strains from the *Enterobacteriaceae*. New Biotechnol..

[118] Leja K., Samul D., Drożdżyńska A., Myszka K., Juzwa W., Pawlicka J., Czaczyk K. (2014). Hypothetical Glycerol Pathways of Newly Isolated Strains Capable of 1,3-Propanediol Production. Acta Biochim. Pol..

